# Whose crisis? Pandemic flu, ‘communication disasters’ and the struggle for hegemony

**DOI:** 10.1177/1363459319886112

**Published:** 2019-11-20

**Authors:** Kevin Hall, Meike Wolf

**Affiliations:** Goethe University Frankfurt, Germany

**Keywords:** biocommunicability, Germany, health emergency, influenza vaccination, pandemic planning

## Abstract

Public health authorities in Germany regard communication as a crucial part of infectious disease prevention and control strategies. Communication becomes even more important during public health crises such as pandemics. Drawing on Briggs and Hallin’s concept of biocommunicability, we analysed the German National Pandemic Plan and key informant interviews with public health experts, critical infrastructure providers and ambulance services. We examined the projected expectations towards the behaviour of the audiences and the projected ways of information circulation informing public health communication strategies during a pandemic. Participants shared the expectation that the population would react towards an influenza pandemic with panic and fear due to a lack of information or a sensationalist media coverage. They associated the information uptake of their target audience with trust in their expertise. While our informants from public health conceptualised trust in terms of a face-to-face interaction, they sought to gain trust through transparency in their respective institutional settings. Our analysis suggests that this moved health information into a political register where their medical authority was open to debate. In response to this, they perceived the field of communication as a struggle for hegemony.

## Introduction

If the Federal Government gets its way and 35 million people get vaccinated, it is to be expected that 8–9 million citizens will suffer from chronic fatigue, fibromyalgia and so on for the next decade.^1^

General practitioner

During the H1N1 influenza pandemic in 2009, a circular email challenged the governmental vaccination campaign against the pandemic strain. The email claimed that the pandemic vaccines Pandemrix and Focetria caused Gulf War Syndrome. The email’s author, a general practitioner, blamed the adjuvant squalene of causing a number of heterogeneous symptoms, such as chronic fatigue, fibromyalgia, headaches and attention deficiency disorder. In a very urgent tone, the author sketched a scenario in which the burden of disease due to the pandemic vaccine would dwarf the effects of the pandemic itself. The circular email spread swiftly and became famous at a national level in Germany. Simultaneously, print media reported about two different vaccines for different population groups. Due to contracts of the Ministry for Domestic Affairs (*Innenministerium*) the vaccine Celvapan from Baxter was provided exclusively to the armed forces (*Bundeswehr*) and members of government (*Bundesregierung*) (Hackenbroch and Traufetter, 2009: 141). The other vaccine, Pandemrix from GlaxoSmithKline, was meant for the general public and contained components whose side effects were fiercely debated. During the pandemic, coverage of vaccine-related topics and events was extensive. Especially, the organisation of the vaccination campaign, the topic of prioritisation of certain groups for vaccination and individual decisions concerning the vaccination by members of government were hotly debated (Deutsche Presse Agentur (DPA), 2009: 11; Frankfurter Allgemeine Sonntagszeitung (FAS), 2009: 1; Hoischen, 2009: 3). This panoply of events together produced an atmosphere that was poorly conducive for the ongoing public health vaccination campaigns. Thus, public health authorities unanimously complained about what they termed a ‘communication disaster’ that had emerged simultaneously with the unfolding pandemic (Krause et al., 2010: 516).

Due to the perceived communication disaster, public health authorities together with the German Ministry of Health agreed to pay more attention to the issue of communication in the revision of the National Pandemic Plan. The result was a plan comprised of two parts. The first part was authored by the Assembly of Ministers of Health (Gesundheitsministerkonferenz der Länder (GMK), 2016). The second part contained the scientific background information and was authored by a committee of experts under the direction of Germany’s disease control agency, the Robert Koch Institute (RKI) (2016). The National Pandemic Plan defines goals and their respective procedures, as well as responsibilities of actors within the state. The following four goals are identified: ‘the reduction of morbidity and mortality within the population; ensuring treatment of sick persons; continuity of essential public services; timely provision of reliable information for policy makers, professionals, the public and the media’ (GMK, 2016: 8). It describes information flows and decision-making structures across different governmental and international levels, surveillance measures, diagnostic capabilities, vaccination campaigns and communication strategies.

In our anthropological study on urban pandemic planning conducted among public health authorities, emergency planners and medical experts, as well as journalists in Germany and the UK, we observed a shared concern for health communication among all our interview partners. Typically, operative responses to public health crises were evaluated positively, whereas communication efforts were seen as either lacking or failing. We take this juxtaposition of preparedness capabilities as an entry point to engage with public health communication. The narrative of the communication disaster hints at the projected ways in which information is circulated and the expected behaviour of the audiences of communication. Rather than discuss the outcome of the public health campaigns, we draw on Briggs and Hallin’s (2016) concept of biocommunicability to analyse the conceptualisation and practice of public health communication.

In their study on public health coverage, Briggs and Hallin (2016) investigated the co-production of knowledge about biomedicine and its coverage in the media. Journalists, scientists, health workers and lay persons all form part of an actor network complicit in the way that health news is narrated. Practices and technologies of communication are central to the way that biomedicalised subjects and objects are represented and constructed. From their extensive material, the authors developed the concept of biocommunicability to describe the performative way by which health coverage not only imparts knowledge but also projects models about how phenomena come to be, how knowledge about them circulates and who should attend to them and how.

They take this sensitivity from actor network theory where the term translation describes the association of actors with other actors. Translation consists of four moments. The moment of ‘problematization’ describes how one actor defines a problem and a possible solution. ‘Interessement’ refers to the process by which the first actor interests others in the identities, roles and functions they would fulfil in the solution to the problem definition. ‘Enrolment’ occurs when the actors actually fulfil their designated roles and associate in order to achieve the common agenda. The moment of ‘mobilisation’ describes the actual mobilisation and exchange of things between associated actors that is necessary to stabilise the network (Callon, 1986). Important for our understanding of the concept of biocommunicability is that, similar moments are passed when one actor wants to convey health messages to an audience. Briggs and Hallin identified three models of biocommunicability that offer different problem definitions and solutions to how health should be communicated: the biomedical authority model, the patient–consumer model, and the public sphere model.

Health communication in the biomedical authority model conceives communication as a unidirectional flow of information. Briggs and Hallin (2010: 151) liken this model to the relation between patient and family physician where the patient’s ‘proper role is to trust and obey’ their doctor. Knowledge production takes place in a supposedly objective, highly specialised and technical sphere. In contrast, other spheres are projected as being dominated by populist, relativist or democratic communication ideologies. Biomedical authorities fulfil the role of the sole reliable sources of health information. Journalists participate in this model by translating health knowledge into popular discourses. The lay public is projected as receiving this information passively. Importantly, due to the necessary process of translation, which is mainly undertaken by journalists who are not part of this elite community of biomedical professionals, health coverage is always perceived as a distortion of the actual information (Briggs and Hallin, 2016: 25–30).

While Briggs and Hallin take many examples for the medical authority model from their material on the ‘swine flu’ pandemic they do not seem to connect the two other models of biocommunicability to disease outbreaks. According to Briggs and Hallin (2010: 152) the patient–consumer model is the most prevalent model in most areas of health communication. It aligns well with conceiving of health services as a market with active, information seeking, medically literate patients making their own independent decisions. In this configuration, the physician takes the role of an informed adviser who works together with her patient/client to find the best solution. As a necessary prerequisite, the client has to be imagined as endowed with sufficient financial resources (Briggs and Hallin, 2016: 33–39). Like in the biomedical authority model, biomedical professionals are the primary source of medical knowledge; but the patient–consumer model projects the patient as an active seeker of information. Both models have in common that they define the function of health information in terms of how ‘it helps the individuals to regulate their behavior in the interest of their own health’ (Briggs and Hallin, 2010: 152).

The third model outlined by Briggs and Hallin is the public sphere model. Here, the usefulness of health information lies primarily in helping citizens and policy-makers ‘make collective decisions about the public interest’ (Briggs and Hallin, 2010: 152). In this model, the audience is addressed as observer-citizen who has to make a judgement about collective decisions and social values. Rather than a linear flow of information from medical experts to a lay public, controversy is framed as conflict between different stakeholders or harmed citizens (Briggs and Hallin, 2016: 39–40). Health information is open to debate. This is particularly the case when health information refers to areas ‘that involve the state, state funding, regulation and policy’ (Briggs and Hallin, 2010: 153).

Drawing on Briggs and Hallin’s concept of biocommunicability we analyse the projected model of the audience and the circulation of information in the National Pandemic Plan and our interviews with experts from public health, hospital staff, ambulance services and critical infrastructures involved in the implementation of emergency plans in their respective area. More specifically we ask: What are the main problems addressed by public health communication during the pandemic? How are the problems framed? Who are the main audiences targeted by public health messages? And what behaviours are expected of them?

## Methods

The article, arguing from the perspective of cultural anthropology, is based upon a 4-year multi-sited ethnography of pandemic preparedness as it is currently practised in many European metropolises. The study builds upon 67 semi-structured qualitative expert interviews, document analysis and participant observations of emergency exercises and meetings between public health authorities and hospitals. We approached experts from local, regional, national and international health authorities as well as people working within virology labs, the media, blue light services, hospitals, airports, public transport organisations and other institutions commonly referred to as ‘critical infrastructures’ (as defined by the European Council Directive 2008/114/EC). While the study builds upon a comparison between urban preparedness in London and Frankfurt, in this article we will focus only on a subset of 26 in-depth interviews conducted in Germany comprising 1–3 participants (*n* = 31). Participants were from ambulance services (4), armed forces (1), critical infrastructures (5), education sector (2), federal emergency management (1), media (4), hospital staff (7) and public health agencies (7).

We conducted the interviews between April 2012 and June 2015. Participation in the study was voluntary and organised around the principles of informed consent. The interviewees were informed about the nature and the purpose of the study as well as about its financing bodies; they were given the right not to answer a question and the opportunity to withdraw from the interview at any point. Their identities were kept strictly confidential. Personal information and empirical data are stored securely. The interviews focused on the participants’ role in pandemic preparedness, the areas of cooperation with other actors in the field, the historical development of pandemic planning, their experience of the 2009 pandemic and how they conveyed information to other actors or where appropriate the public.

The interviews were transcribed verbatim using the transcription software f4. Data analysis was conducted using ATLAS.ti software. Interviews were coded independently by both researchers. Codes were then discussed and common themes (e.g. panic and fear, channel of communication, target audience) identified and aggregated into categories (e.g. cause of fear, communication strategies) (see Corbin and Strauss, 2008; Glaser and Strauss, 2008). The interviews were then carefully reread to identify directionality of information flow, roles in information production and uptake and target audience-specific communication strategies.

This article does not aim to present an exhaustive overview over the manifold and complex empirical data collected throughout the 4 years of study. For example, participant observation data of emergency exercises and public health meetings are not included here but informed our understanding of the locations and circumstances of information flow between actors. Instead, only those interviews were chosen from the German subset concerning the broader issues of circulating and managing information, its underlying frictions and controversies, as well as rationales and mindsets that informed the crafting of public health messages.

## Results

We present two major themes in communication that emerged from our analysis. All actor groups shared in one way or another a certain concern with a public in panic. Panic was associated with certain projected behaviours. Importantly, not all of these behaviours were observed during the H1N1 Pandemic of 2009. They represented virtual possibilities of an acting public. Thus, in a first step we analysed how public health officers and medical staff perceived the public and what behaviours they expected. In a second step, we discuss the explanations our participants offered in regard to the perceived ‘communication disaster’ associated with the vaccination campaign outlined in the introduction. Our analyses centres on how our participants framed the failure and what they thought would have been an appropriate communication strategy to prevent this ‘disaster’ from happening again.

### The public in panic as a scenario

Throughout the interviews, participants articulated a concern that the outbreak of a pandemic could cause the public to panic. Panic was linked to crowds and a sense of fear. While most participants referred to panic, fear and even hysteria simply as a reaction to the outbreak itself, some participants offered additional causes for the emotional state of the public. Among these causes, a lack of knowledge and media coverage were mentioned most frequently. For example, public health officer E who was involved in the first draft for a German pandemic response plan and was now coordinating pandemic response plans in a German federal state linked panic to rising case numbers. When we asked her to delineate the most important agencies within the federal state with whom she collaborated in pandemic planning she first mentioned the local and regional public health authorities and the politicians. After this she moved on to depict the importance of collaboration with the federal state’s Ministry of Education and Cultural Affairs as well as the Ministry of Justice.

You would first notice the problem in the Ministry of Education and Cultural Affairs. They are responsible for the schools. In our Ministry [Ministry of Health] we have the kindergartens as well. And in such a case the children fall ill first and most frequently. And then everybody’s in panic. And then schools are closed and oh my God. So we have had a relatively close communication and surveillance in this area. How many sick people do we have in the schools? And so on. But you’ve also got other closed areas like the Ministry of Justice who have their prisons. They are, so to speak, a closed circuit. But you have to look there as well. Are they developing their plans? Have they made their plans? Are they correct? And then, of course, you have, let’s put it like this, with the rest, our contact is broader. But the fear and the hysteria which erupts there, is everywhere (Public health officer E, August 2014).

The H1N1 pandemic of 2009 had introduced a new concept of pandemic into public health preparedness planning. Up until 2009 a pandemic was expected to entail severe morbidity and many deaths. However, the 2009 pandemic was referred to by some of our participants as mild. Clinician B is responsible for hospital hygiene and infection control plans. To him this new type of pandemic was challenging because it overburdened primary care facilities with people who were only afraid of being infected.

Interviewer: What are the biggest challenges during an influenza pandemic?

Clinician B: I think, one has to distinguish between a pandemic that has a low morbidity and one that has a high morbidity. In the low morbidity case, I think, it is decisive to quickly clarify, well, there is a pandemic development but strictly speaking, it [the pandemic] proceeds very mildly. So, to keep the panic in the population low; or what is panic? Well, the fear in the population. To put something against it and through this to keep the burden on the medical structures at a low level. If you’ve got one [a pandemic] with a high morbidity, then the challenge is public life, so to speak. This not only affects hospitals. All areas of life are affected and have to be kept operational (Clinician B, May 2013).

Clinician B is concerned that although the pandemic is mild, the hospitals would be crowded with scared people who were not actually ill, the so-called worried well. Both clinician B and public health officer E share the belief that along with the actual public health crisis the emotional state of the population has to be managed as well. Scared crowds were also associated with danger. A scenario frequently turning up in connection with epidemics and pandemics in fiction are violent mobs, or rioting people pillaging supermarkets and pharmacies. We encountered this fictitious scenario in the field when public health officer E told us about the expectation of raids on vaccine transports during the pandemic of 2009 in Germany.

Interviewer: It’s interesting that you mention the concept security in this context. That’s something we have asked ourselves. Is pandemic planning essentially about security? Or is it about health?

Public health officer E: In our Ministry it is health. That is, to enable the survival of as many people as possible. Security always comes into play – what I’ve mentioned earlier; is the system breaking down? Are we heading towards a catastrophe? Are the financial authorities – very important – unable to operate. Then security comes into play. But, as I said earlier, actually the idea is, that as many [people] as possible consider, so that we don’t even get into a catastrophic situation. But we can’t rule it out. Because if the population is afraid and in 2009 they were, extremely so, although it [the pandemic] wasn’t that severe, then it will be hard to control the hysteria.

Interviewer: How would you control it? You have mentioned communication as a medium already.

Public health officer E: Yes. Precisely. That’s essential. As I said, clear propositions, clear announcements are ultimately the only thing you can do. Under certain circumstances you have to reckon with riots. Of course, we did. So it was reckoned with all sorts of things. One could raid vaccine transports. Do we need police protection for the vaccination sites? Do we need police protection at the pharmaceuticals issuing office and so on? Of course (Public health officer E, August 2014).

Public health officer E perceives the mass psychological dynamics of crowds as potentially dangerous and linked to a security problem that can ensue when communication fails to ‘control the hysteria’. Another disruptive effect of crowds that participants mentioned were too few people turning up at their workplace. Medical staff and public health officers described the effects of fear on their staff. Regarding the question of how hospitals recruited more personnel to manage the higher number of patients clinician B answered:

You haven’t got more staff, that is. That’s not the question. There’s a completely different question at stake, if it really is dangerous, [short pause] Are your staff turning up? So what are your plans so that your personnel actually come to work. It’s generally known about New Orleans. Public life completely collapsed there for that reason, among others. Because all of a sudden, personnel in public services didn’t come to work anymore. SARS in Canada. They had a huge problem because people didn’t want to go to work anymore. So imagine, I work here and my wife at home says: Are you out of your mind? You’ll pick up the deadly epidemic at the hospital. You’re staying at home. You’re not going there (Clinician B, May 2013).

This was a prevalent topic across all fields of work: Fear of catching the flu at work. A public in panic was perceived as the cause for a range of problems related to the number of people turning up at one place – too many or too few. Most of our participants from public health and the clinic defined fear as an outcome of insufficient and/or faulty information. For instance, nurse A who was responsible for training staff for work in the isolation ward explained that his colleagues felt uneasy working with infectious diseases at first.

Interviewer: Do you have the impression that working in this area provokes anxiety in nurses?

Nurse A: You just need regular information and training. Then there’s no fear. Colleagues start off having fear, that’s something you notice during information sessions, when we recruit new colleagues. But that’s mostly so called nescience. It’s just that. It’s only nescience (Nurse A, April 2013).

Participants in our study regarded the knowledge deficit about infectious diseases not only as a deficit of the general public. They also attributed it to medical staff and employees in the public administration and critical infrastructures. Behaviours such as staying away from work, forming crowds in the accident and emergency department, or not getting the flu shot were often interpreted as a result of this lack of knowledge. However, this knowledge void could be filled apparently easily by providing colleagues or the public with good information. Public health officer D was involved in revising the National Pandemic Plan. As a former paediatrician he compared communicating with the public to the situation in the surgery:

But I think the individual needs reliable sources and a good basis. And then, I’m convinced, will he, fathers with their children – they will take a responsible stance, I think. That’s my personal experience. So, if I have provided the parents with good advice, they can work with that. And this will already have positive effects on the child. So, if I reassure the parents, then the child will be calm as well. That is, reassuring in the sense of providing meaningful information so that they know what they can do, what they have to do. And that’s here – That’s why communication is so important (Public health officer D, March 2014).

Public health officer D likens communication to the relationship between patient and family physician and links information and the provision of clear options for action to the management of fear. Public health officer A, who was involved with urban pandemic planning gives another example for the construction of a linear causality between knowledge, health behaviour and emotional states. He had conducted a survey among airport passengers and staff returning from or departing to Mexico just at the beginning of the pandemic.

And it’s very interesting what we found. That is, well it’s a study that cannot so easily be transferred to the conditions here [in the town]. But in principle it can. Because we found: The better the population is informed, the tourist or whoever, the less afraid he is, and the more adequately he reacts, and the better one can deliver one’s message. And the less he is informed, the more he besieges your A & E and ‘steals your time’, in inverted commas, although you know for sure: he can’t be ill. But he is frightened. So you have to take care of him, understandably (Public health officer A, November 2012).

This view was also present in the revised National Pandemic Plan authored by a task force of experts from public health and medical associations (RKI, 2016: 218–220). It conceptualises risk communication as a long-term pedagogical project to shape acceptance for public health measures against the pandemic. It states:

Risk communication is a (often long term) project that, referring to health risks, informs about the relationship between health-related behaviour and the resulting adverse health effects, harms, or diseases. The primary goal is to improve the knowledge of the population regarding health risks and their impacts. [. . .] From the understanding of risks and the associated realisation of the potential self-endangerment, a willingness to change or adapt behaviour should follow by way of changing one’s knowledge and attitudes, so that risky health behaviour (e.g. smoking, neglecting vaccination) is abandoned or actions are taken up to avoid health risks (e.g. giving up smoking, protecting against passive smoking, preventive vaccination against influenza) (RKI, 2016: 188).

The plan imagines communication as a direct transmission of knowledge to action unmitigated by individual contingencies, motives and values underpinning health behaviours. While this model of communication is informed by a rational actor model, in our interviews the public and its behaviour were conceptualised as part of a scenario with defined reactions to ‘good’ or ‘bad’ communication. Furthermore, in our interviews the prevalent actor model was not so much a rational actor but a population that our participants from public health and the medical sector perceived in their affective qualities. For public health officer E in the quote above a public in panic is the result of a failure of communication efforts to ‘control the hysteria’. In the next section, we turn to the explanations for the failure of communication offered by our participants.

### The struggle for hegemony in communication: fighting mistrust with transparency

Many of our participants were concerned about how the vaccination campaign and the public discussion about it had undermined their own efforts to promote vaccination. For example, ambulance officer B contrasted his staff’s reluctance to receive the flu jab with their overall vaccination status.

Interviewer: What is your assessment on the vaccination status of your staff?

Ambulance officer B: The uptake isn’t as high anymore. I have surveyed this during the hygiene training this year. The uptake with respect to the influenza vaccination is very low. With respect to the other infectious diseases like hepatitis A and B my colleagues are very well informed. They even know their titre and nearly 100 percent accept the vaccination offer and know about the threats of this disease. With respect to the influenza vaccination however, we’ve had a break because of the swine flu slash new influenza. And in relation to the squalene problematic with the two vaccines and also due to the media coverage the uptake could not be maintained. And as a specialist for hygiene it was difficult for me to convey, that an influenza vaccination was necessary now to keep the ambulance service operational. But my colleagues were consternated by the supply of two different vaccines. There was talk about a VIP vaccine and a commoner vaccine of lower quality. Later the authorities and the media explained the situation and classified it as safe. But that was closing the stable door after the horse had bolted (Ambulance officer B, March 2013).

The so-called commoner vaccine contained the adjuvant squalene supposed to make the vaccine more effective and traces of mercury as a preservative. The German government had ordered another vaccine without these components. In October of 2009, just before the start of the vaccination campaign the media covered this story extensively with conflicting information on who would receive this other vaccine (e.g. Seidler, 2009). The presence of a vaccine without adjuvants and preservatives for a selected group in Germany led to a discussion about the safety of the so-called commoner vaccine in the media. Public health officer D expressed his surprise about this discussion. For him everything had worked according to the published plan.

The vaccines required the time outlined in the plan. Approximately six months. Which was the optimal time stated in the plan if you start at zero up until you are ready to distribute it. But the communication about it and the communication of what we did and did not know at the time, that worked relatively badly. This opinion was collectively shared. Much of what was known, or could have been known, was simply not known. On the other hand much of what was not known was underestimated. And this resulted in doubts and insecurity which were responded to rather reactively. Especially concerning the vaccine or components of the vaccine. And that the plan was not read. It’s in there why the vaccine contains adjuvant and which adjuvant and for what. If you look it up you can see it. It’s in the chapter of the 2007 plan on the table in front of you. But this was not communicated. We probably should have been more active in communicating this at the time (Public health officer D, March 2014).

According to public health officer D much criticism of the components of the pandemic vaccine could have been mitigated by explicitly communicating the ingredients before the vaccination campaign. Knowledge about the components of the vaccine is expected to create acceptance of the vaccination campaign. We call this communication strategy *acceptance through familiarity*. Here, the communicating person anticipates a knowledge gap in the audience. This strategy targets an audience that is expected to react uncooperatively towards an unexpected measure. By familiarising the audience with the measures the knowledge gap is mended and cooperative behaviours are thought to become more likely. Clinician A mentioned another instance of this strategy when he spoke about how journalists could be convinced to produce a less sensationalist coverage.

Interviewer: Is there anything we haven’t touched upon in our interview, that you deem important?

Clinician A: External communication. The media. Because here’s what I think. In 2009, 2010 but also already during avian flu, it’s what the population knows. This is called the Hörzu-Effekt.^2^ If this or that is printed in Hörzu, the surgeries are crowded the next day. This is valid of course also for these kinds of situations. Naturally, the Yellow Press [uses English word; refers to tabloid press] values it as a sales hit. But there are immediate consequences for patient care when a topic like that emerges. And you can prospectively sit down with journalists. You can give them guided tours. You can discuss emergency plans, not in detail but their general idea. So that they at least know the activities it contains. That improves the acceptance of the measures or reduces the fear of your colleagues as well. That is the decisive point. When I show, that I am prepared then more colleagues show up to work (Clinician A, January 2013).

Three participants working in the area of contingency management in companies that provided electricity, gas and water expressed their frustration with the prioritisation of certain sectors in the vaccination campaign. They defined themselves as critical infrastructure providers. Public statements by the health authorities had mentioned that critical infrastructures would be prioritised along with medical staff, police and the fire brigade. They therefore planned for vaccination campaigns in their companies. But when the vaccine became available they did not at first receive any vaccine.

Well, there is a guideline from the Federal Government or some Ministry of Health at the state level. There are prioritised groups which are vaccinated first during a pandemic. Naturally doctors and hospital staff along with the fire brigades, police and ambulance services are first, I think. I said, we provide the infrastructure for electricity and gas. And our people, not all of them, not administration, but the people who are responsible for keeping the electricity flowing, who are essential for preventing a blackout, like the 15 people in the control stand. If they’re not there nobody monitors the mains and the gas grid anymore. Everything breaks down. I said, these people are at least as important as a nurse. Nobody in the local public health authorities accepted that. It seems that in Germany policymakers have not understood, that power and gas supply are part of the critical infrastructure (Critical infrastructure B, December 2013).

Two participants from critical infrastructure A told a similar story about their efforts to receive vaccines. Critical infrastructure A1 criticised the contradictory messages communicated by different governmental levels.

Interviewer: From your perspective, what are the key issues that have to be solved for future pandemics?

Critical infrastructure A1: Well, what I think is simply, that communication of the Federal Government, the federal state government and the communal government, that it is properly carried out, that they do not speak with different voices. That on the part of the state communication of provisions is coordinated better. Because in my opinion that fuss that occurred during the swine flu, not only with respect to our staff, but also with respect to the population, caused uncertainty. And the media were grateful to cover it. And we all still remember the headlines (Critical infrastructure A1, September 2013).

From an audience perspective critical infrastructure A1 calls for a *one-voice policy*. He frames communication as a problem of political leadership. The different messages suggested a split leadership open to contest. A one-voice policy in his mind could serve two purposes. It prevents uncertainty in the population about what to expect and what to do and it prevents the media from undermining the communication campaign. This strategy is central to the revised National pandemic plan mentioned earlier. For example, it states:

Due to the federal system in Germany and the different areas of jurisdiction between Federal Government, federal state and communal government it entails, for infection control and in (pandemic) crisis management it is inevitable, that the public and the media are addressed and spoken to by different official authorities. By coordinating procedures among authorities on the Federal and the state level it is to be ensured, that conveyed messages and recommendations are consistently given on the same information base and are not in contradiction to one another, so that emergence of uncertainty in the population and loss of trust in crisis management and crisis communication of public authorities is avoided. This requires a fast procedure for information delivery and coordination, so that preferably all authorities communicating with the public act upon the same information base and convey consistent messages (RKI, 2016: 195).

In this excerpt retaining the public’s trust emerges as a third goal of the one-voice policy. The agencies of public health officers C and D were involved in the evaluation of the scientific knowledge that informed the National Pandemic Plan as well as actual policies during the pandemic. Throughout their interviews they repeatedly emphasised that the information base for decisions was made transparent by publishing them. Public health officer C gave a powerful account of this practice when he described the process of evaluating the pandemic of 2009.

We had a relatively intensive phase of lessons learnt after the pandemic during which we tried to evaluate what had happened during the pandemic. What can be learnt from it. How can it be improved. You probably know about the workshop that was organised by the Robert Koch Institute. We were present. The results are published in the *Bundesgesundheitsblatt*. The results of the workshop can be looked up there. We recently attended a hearing at the health committee where we were asked about the preparations for the pandemic and what we had learnt and what could be improved. It can be checked. It should be published on the webpage of the Bundestag. That means, these are completely public, transparent procedures in which it is attempted to process what was learnt and update pandemic plans and be prepared should a pandemic occur again (Public health officer C, May 2013).

The information base is framed here as part of a democratic procedure. Demonstrating transparency by publishing processes and the basis of decision-making is directed towards a public that has the right to know and hold officials accountable for political decisions.

This framing of public health communication is, however, complicated by a mistrust towards the media we repeatedly encountered throughout our interviews. The way the swine flu pandemic in general and the vaccination campaign in particular were covered was criticised by our participants throughout the examples presented here. And participants from public health as well as clinicians saw the need to actively manage the media like in the example given by clinician A above. The National Pandemic Plan cited an assessment by the Federal Institute for Risk Assessment (*Bundesinstitut für Risikobewertung*) stating:

The pre-crisis phase lends itself to fostering contacts to such media and consumer groups with whom collaboration in the event of a crisis is possible without excessive hysteria (RKI, 2016: 191).

The term ‘excessive hysteria’ hints precisely at the underlying assumption that the public and the media are always somewhat hysterical. Also the treatment of dissenters, like self-proclaimed experts or critics of the vaccine like the doctor whose email about the possible side effects of the vaccine we quote in the epigraph complicate an idyllic framing of public health communication as a democratic, transparent endeavour. Public health officer A sketched his communication ideal in regard to the dissenting doctor in saying:

Public health officer A: So this is an essential part. First, the information. In principal that’s much more important for us now than any other infection control measures. For me risk communication is much more important.

Interviewer: Directed at the population?

Public health officer A: Directed at the population. But not only. The fire department and the other departments as well. Administrative staff working in human resources and purchasing department, they are as frightened as the normal population. That’s for sure. We try to establish our agency as a firm anchor for the population. So that they can say: If they say that, that will be at least half right. If I achieve this until I retire then I’ll have achieved quite a lot. So, in principle, one should be considered as an independent source of expertise. So that someone like this general practitioner unfortunately up to mischief [. . .], that she doesn’t get a chance. She can send the email but people say: that’s ridiculous stuff she’s writing (Public health officer A, November 2012).

The utopia expressed in this quote is one of hegemony in communication. False or non-conforming information in circulation would not have to be responded to because the general public would ignore it altogether, thus rendering that piece of information irrelevant. In this quote public health officer A structures the field of public health communication as constituted by two opposed parties: dissenters like the general practitioner who do not share the belief in the necessity of the vaccination campaign, and those who believe in the necessity of public health campaigns. The problem dissenters pose to public health campaigns was also recognised by the emergency planners involved in the revision of the National Pandemic Plan. In the chapter on communication they put forward a strategy that constitutes the field of communication in terms of a struggle for hegemony, which should specifically shift the balance in their favour:

During the acute crisis a quick and open communication aims at various goals: ‘it is the path to gain hegemony of communication in public opinion, it can forestall rumours and it will make the interest taken by the media decline if it can be convincingly assured that all relevant information is openly and actively communicated’ (RKI, 2016: 192–193).

Interestingly, here the media, which are typically appreciated for their role in democratic deliberation and as one of the most important channels of communication, become a nuisance factor for crisis communication. The media have to be fed information in such a way that they lose interest in providing additional information beyond what they are supposed to relay to the public. Communication is framed as a struggle for public opinion among various societal actors that is fought and won by deploying the communication strategies described here.

## Conclusion

Our informants from public health and hospitals, as well as the German National Pandemic Plan, conceptualise the general public as scared, potentially overreacting and panicked during a pandemic. This very much mirrors the results of Davis et al. (2011) on the problematisation of the general public in Australian pandemic control documents. In the German case, participants constructed the panicking public as a mass phenomenon that was disruptive of processes in two ways: either as a turning-up of too many people forming crowds at the wrong places, or as a turning-up of too few people at the right places, such as their workplaces. Participants from public health and hospitals attributed fear or panic to a lack of knowledge in the public as well as their colleagues. Communication strategies would have to target this affective register of the population. Our participants framed their strategies towards this register in terms of ‘calming’, ‘familiarising’ and ‘understanding risks’.

‘Calming’ and ‘reassuring’ the public was mentioned by public health officer D in the context of a patient–doctor relationship. This relationship as Briggs and Hallin (2010: 151) point out built on the patients’ trust towards their family physicians. When patients trust their doctor, they stop looking for alternative options for action. In the words of public health officer D, they know ‘what they have to do’. This is the prototypical relationship of the biomedical authority model of biocommunicability. However, this face-to-face relationship of trust is transformed in the context of crisis communication by official actors. Participants from public health interpreted trust in the sense of transparency. Transparency of procedures and the publishing of decision bases, as well as admitting limitations to knowledge, constituted public health authorities as accountable and addressed the public as citizenry. Addressing an audience as citizens moves health communication into a political register. Briggs and Hallin (2010: 152) refer to this mode of biocommunicability as the public sphere model. In this model health information becomes subject to open debate.

In our interviews with health professionals both models, the biomedical authority model and the public sphere model are present. Sometimes they come into conflict with one another, especially when participants talked about the vaccination campaign during the pandemic. Public debate revolved around belonging to a certain social class symbolised either by receiving a vaccine deemed safer or being part of a prioritised group. In this context one of our participants framed communication in a way similar to what the National pandemic plan calls a ‘hegemony of communication in public opinion’ (RKI, 2016: 192–193). Ernesto Laclau and Chantal Mouffe (2014 [1985]) have transferred the Gramscian notion of hegemony to the analysis of discourses. Hegemony as a discursive concept describes the prevalence of certain patterns of production of social meaning (Laclau and Mouffe, 2014 [1985]: 91). From this perspective the debate about the safety of the vaccine was organised around the category of trust in both its political and affective registers. Brown and Calnan (2010) make a similar observation regarding the pharmaceutical industry where the perceived safety of drugs is often linked to trust in the manufacturer. Perceiving the vaccine as safe was in the eyes of the public health officers as much a matter of the public’s understanding of science as of its mistrust in the sources of the information. In terms of the actor network perspective of biocommunicability hegemony is achieved by one actor if the actors it wishes to enrol (e.g. journalists, the public) recognise the framing of the issue (health or politics) and fulfil the roles assigned to them in the communication of health information.

Similar to the findings of Wagner-Egger et al. (2011) blaming the media but also blaming dissenters for the low uptake of vaccination helped some of our participants to make sense of the ‘communication disaster’. Journalists are arguably the first audience when health authorities want to relay messages to the public. Framing the media as adversary rather than partner might impinge on the readiness to answer journalist’s questions. One of our participants proposed to invite journalists to emergency exercises or meetings to discuss public health strategies with them. Briggs and Hallin (2016: 120–124) emphasise the role that journalists’ participation in exercises played for the production of the media coverage of the H1N1 pandemic within the first 24 hours of its detection. Their account sounds a note of caution in regard to how too much familiarity with exercises influences journalists to write stories about actual outbreaks that resemble the rehearsed scenario.

What is apparent from our findings is that health professionals underestimated the political register of the vaccination campaign. Coverage of issues of funding, prioritisation and safety of different vaccines followed the routines of political reporting opening the campaign to legitimate controversy. In addition, the dominance of the patient-consumer model might also complicate communication during a public health emergency. While outside of a crisis situation patients may actively seek information in order to maximise their health using all sources at their disposal, during the crisis public health authorities felt that they had to compete with these sources. In our interviews the one-voice policy emerged as a means to reduce the circulation of contradictory information from official sources. But this policy cannot (and should not) prevent controversy altogether. Our results as well as the recent debate about mandatory vaccination for measles for children in schools and nurseries suggest (e.g. Oltermann, 2019), that the struggle for hegemony with respect to vaccination campaigns in Germany, at least in part, seems to be centred on the question of whether vaccination is negotiated according to the rules of medical sciences or politics. If this is the case then it would be prudent to actively foster public debate about vaccination rather than waiting for the next pandemic to occasion the debate.

## Notes

1. All translations from German are our own.

2. Hörzu is the first German television and radio listing magazine that was published after the Second World War. The name translates as ‘Listen!’. Over the years, the magazine focussed more on yellow-press topics and style than on information on television and radio listings.
